# Transcriptional responses to biologically relevant doses of UV-B radiation in the model archaeon, *Halobacterium *sp. NRC-1

**DOI:** 10.1186/1746-1448-4-13

**Published:** 2008-08-29

**Authors:** Ivan Boubriak, Wooi Loon Ng, Priya DasSarma, Shiladitya DasSarma, David J Crowley, Shirley J McCready

**Affiliations:** 1 Department of Biochemistry, University of Oxford, South Parks Road, Oxford OX1 3QU, UK; 2 School of Life Sciences, Oxford Brookes University, Gipsy Lane, Oxford OX3 0BP, UK; 3 Center of Marine Biotechnology, University of Maryland Biotechnology Institute, 701 E. Pratt St., Suite 236, Baltimore, MD 21202, USA; 4 Molecular and Structural Biology Program, Greenebaum Cancer Center, University of Maryland, Baltimore, MD 21201, USA; 5 Natural Sciences Department, Assumption College, 500 Salisbury Street, Worcester, Massachusetts 01609, USA; 6 Institute of Cell Biology and Genetic Engineering, UAS, 148 Zabolotnogo Street, Kiev, 03143, Ukraine

## Abstract

**Background:**

Most studies of the transcriptional response to UV radiation in living cells have used UV doses that are much higher than those encountered in the natural environment, and most focus on short-wave UV (UV-C) at 254 nm, a wavelength that never reaches the Earth's surface. We have studied the transcriptional response of the sunlight-tolerant model archaeon, *Halobacterium *sp. NRC-1, to low doses of mid-wave UV (UV-B) to assess its response to UV radiation that is likely to be more biologically relevant.

**Results:**

*Halobacterium *NRC-1 cells were irradiated with UV-B at doses equivalent to 30 J/m^2 ^and 5 J/m^2 ^of UV-C. Transcriptional profiling showed that only 11 genes were up-regulated 1.5-fold or more by both UV-B doses. The most strongly up-regulated gene was *rad*A1 (vng2473), the archaeal homologue of *RAD51*/*recA *recombinase. The others included *arj*1 (vng779) (*rec*J-like exonuclease), *top*6A (vng884) and *top*6B (vng885) (coding for Topoisomerase VI subunits), and *nrd*J (vng1644) (which encodes a subunit of ribonucleotide reductase). We have found that four of the consistently UV-B up-regulated genes, *rad*A1 (vng2473), vng17, *top*6B (vng885) and vng280, share a common 11-base pair motif in their promoter region, TTTCACTTTCA. Similar sequences were found in *rad*A promoters in other halophilic archaea, as well as in the *rad*A promoter of *Methanospirillum hungatei*. We analysed the transcriptional response of a repair-deficient Δ*uvr*A (vng2636) Δ*uvr*C (vng2381) double-deletion mutant and found common themes between it and the response in repair proficient cells.

**Conclusion:**

Our results show a core set of genes is consistently up-regulated after exposure to UV-B light at low, biologically relevant doses. Eleven genes were up-regulated, in wild-type cells, after two UV-B doses (comparable to UV-C doses of 30 J/m^2 ^and 5 J/m^2^), and only four genes were up-regulated by all doses of UV-B and UV-C that we have used in this work and previously. These results suggest that high doses of UV-C radiation do not necessarily provide a good model for the natural response to environmental UV. We have found an 11-base pair motif upstream of the TATA box in four of the UV-B up-regulated genes and suggest that this motif is the binding site for a transcriptional regulator involved in their response to UV damage in this model archaeon.

## Background

Solar radiation encompasses UV wavelengths ranging from <290 nm (UV-C, which is filtered out by ozone in the stratosphere), 290–320 nm (UV-B) and 320–400 nm (UV-A) as well as non-UV radiation, including visible (400–700 nm) and infrared (>700 nm). It has been well established that UV-B and UV-C cause mutagenic and cytotoxic damage to cells resulting from the induction of photoproducts in DNA, principally cyclobutane pyrimidine dimers (CPDs), 6-4 photoproducts (6-4 pps), and their Dewar isomer. More recently, it has been shown that CPDs are also the predominant DNA lesions caused by UV-A [1-4]. Most laboratory studies of the responses of living cells to UV have used high UV doses and mainly energy emitted from germicidal lamps at 254 nm (UV-C). However, these studies reflect neither biologically relevant doses nor wavelengths, because UV-C never reaches the Earth's surface and because the doses of UV in natural sunlight are low in comparison to the doses commonly used in the laboratory.

Most organisms have developed multiple strategies for surviving UV radiation. These can include protection from damaging wavelengths, cell cycle arrest, and activation of various pathways for repair of UV-damaged DNA. Tolerance mechanisms, such as recombination and lesion by-pass, which allow cells to survive when lesions remain unrepaired in the DNA are also critical for survival [5]. In consequence of this variety of responses, even organisms exposed to high levels of sunlight in their natural environment show considerable variation in their UV sensitivities [1,6,7]. Among these are the highly radiation-resistant halophilic archaea, such as *Halobacterium *species NRC-1, which are exposed to intense solar radiation in their natural hypersaline environments.

The sequenced model archaeon, *Halobacterium *sp. NRC-1, is highly resistant to the damaging effects of UV light. One reason for this is highly efficient photoreactivation of DNA damage [8,9] but, even in the absence of photoreactivation, *Halobacterium *is significantly more UV-tolerant than *Escherichia coli *or *Saccharomyces cerevisiae *[10,11]. It is not yet clear why this is so. When the genome sequence of *Halobacterium *sp. NRC-1 became available, it seemed that a likely explanation was the existence of multiple repair systems because the genome contains homologues of both eukaryotic and bacterial nucleotide excision repair (NER) genes [12]. However a functional analysis of key repair genes has shown *Halobacterium *sp. NRC-1 depends primarily and possibly solely on a bacterial-type NER, involving UvrA, UvrB and UvrC proteins for repair of CPDs and 6-4pps [10]. There have been suggestions that carotenoid pigments may also play a part in protection of *Halobacterium *from UV radiation [13,14]. Two possible roles for carotenoids are in protecting DNA by directly absorbing UV and thus preventing formation of photoproducts, or in providing energy for excision repair. It has been shown that mutants lacking carotenoid pigments are more sensitive to UV irradiation than wild-type cells [14] and there is evidence for protection of DNA by bacterioruberin *in vitro *[13]. Another interesting observation is the very low occurrence of dipyrimidines in the genome of *Halobacterium *sp. NRC-1 which should result in fewer photoproducts [15,16]. However, a comparison of photoproducts in DNA from UV-irradiated *Halobacterium *and yeast cells has not shown any detectable difference in numbers or types of photoproducts induced, suggesting carotenoid protection and dipyrimidine number are not major factors [17]. Another possible contributor to the high UV tolerance may be the existence in *Halobacterium *cells of multiple copies of the genome, with 15 to 25 copies of the 2-Mbp chromosome per cell [18]. However, although high copy number and its accompanying genetic redundancy might be expected to increase a cell's chances of surviving DNA damage, the relationship between UV resistance and ploidy is not clear-cut at these high copy numbers [19,20]. In *Deinococcus radiodurans *an important factor seems to be that the recombination/repair protein, RecA, plays a critical role in UV tolerance [21], and this may also be the case in *Halobacterium*.

Many of the cellular responses to UV irradiation are constitutive but in all organisms studied to date there are also inducible responses. These have been investigated by a number of groups using whole genome transcriptome profiling. The best studied example of transcriptional regulation in microorganisms is the SOS response in bacteria such as *E. coli*, which involves LexA-dependent up-regulation of about 40 genes, including excision repair genes [22]. In addition, a number of genes, including *nrd*A, and *nrd*B (coding for ribonucleotide reductase subunits) are up-regulated independently of LexA, though mostly not more than 2-fold [22]. Depending on the eukaryote, a variety of genes are up- and down-regulated in response to UV-damage, but no eukaryotic equivalent of the bacterial SOS response has been identified [23].

There have been several studies of transcriptional responses to UV in the archaea [11,24-28]. Although these studies have used different experimental regimes, there are certain common observations, including the absence of a coordinated SOS-like response. A study by Salerno *et al*. [24] suggested that, in *Sulfolobus solfataricus*, the homologues of human repair genes *XPF*, *XPG *and *XPB *(homologues of *Saccharomyces cerevisiae RAD1*, *RAD2 *and *RAD25 *respectively) were UV-inducible. However, this was not confirmed by more recent analyses [25,28] and transcriptome analysis in *Halobacterium*, has not shown excision repair genes to be up-regulated by UV [11,26].

Most laboratory studies of UV damage have used short-wave UV because low-pressure mercury vapour germicidal lamps, which emit at 254 nm, are readily available and they produce essentially the same type of DNA damage as UV-B, the most damaging wavelengths in sunlight at the Earth's surface. They focus on short-wave UV (UV-C) at 254 nm, which is blocked by oxygen and ozone in the stratosphere and therefore is a wavelength that never actually reaches the Earth's surface. Most studies of transcriptional responses to UV radiation have also used UV doses that are very much higher than those encountered in the natural environment. Two archaeal studies used UV-C doses of 200 J/m^2^, a more recent one used 75 J/m^2^, and in our own previous study we used 30 J/m^2 ^and 70 J/m^2^. High UV doses have traditionally been used for studies of repair of photoproducts because the assays for measuring DNA damage are rarely sensitive enough to allow the use of lower doses. However, for transcriptional studies, there is little justification for using doses that are many-fold higher than organisms are ever exposed to under sunlight.

Table 1 shows the amount of damage produced by various doses of UV used in transcriptional studies and shows that a dose of 200 J/m^2 ^(administered over a period of only 1 minute) produces more DNA damage than 12 hours of sunlight [29,30]. It is well known that initiation of DNA replication and transcription are inhibited by UV in a dose-dependent manner, so we believe that high doses are likely to produce artefacts, making it important to use more biologically relevant doses. In order to approach biologically relevant radiation conditions for our transcriptional analysis, we used a broad-band UV-B lamp and low doses of UV, producing equivalent damage (in terms of CPDs) to 5 J/m^2 ^and 30 J/m^2 ^of UV-C. The lower UV-B dose used in this study, equivalent to 5 J/m^2 ^UV-C, induces the same amount of damage (in terms of CPDs) in 30 seconds as about 20–30 minutes of sunlight (see Table 1).

**Table 1:** Induction of CPDs (cyclobutane pyrimidine dimers) by different doses^1 ^of UV-C [47] used in microarray studies compared to CPDs induced by sunlight [49]

UV-C dose	CPDs induced per kb	Duration of UV dose	Reference (Organism studied)
200 J/m^2^	1.67	1.05 min	Baliga *et al*. (*Halobacterium*) [11]
			Gotz *et al*. (*Sulfolobus*) [25]
75 J/m^2^	0.63	not known	Fröls *et al*. (*Sulfolobus*) [28]
70 J/m^2^	0.59	1.16 min	McCready *et al*. (*Halobacterium*) [26]
40 J/m^2^	0.33	1 min	Courcelle *et al*. (*E. coli*) [22]
30 J/m^2^	0.22	0.5 min	McCready *et al*. (*Halobacterium*) [26]
Sunlight for 1 day	0.50	12 hours	Wilhelm *et al*. (^2^DNA dosimeter) [29]
Sunlight for 1 day	1.00	12 hours	Visser *et al*. (^2^DNA dosimeter) [30]

^1 ^Irradiation schemes vary in each case.

^2 ^In a DNA dosimeter, naked DNA is exposed to sunlight and the number of CPDs is measured.

## Results

We irradiated wild-type *Halobacterium *sp. NRC-1 with a dose of UV-B that induces the same number of CPDs per kb DNA as 5 and 30 J/m^2 ^UV-C (we will refer to these regimes as *5 J/m^2 ^and *30 J/m^2^) and irradiated a Δ*uvr*A Δ*uvr*C double deletion mutant, which lacks the capacity for nucleotide excision repair, with a dose of *5 J/m^2^. We have compared the transcriptional response to these UV-B doses and to the response to irradiation with 30 J/m^2 ^UV-C, which we reported previously [26].

### The transcriptional response to a UV-B dose equivalent to 30 J/m^2 ^UV-C

After a UV-B dose of *30 J/m^2^, 103 genes were significantly up-regulated (1.5-fold or above, p-value < 0.001) at 1 hour and/or 3 hours after irradiation. The most strongly up-regulated genes included *rad*A1 (vng2473) (gene for RecA/Rad51 recombination protein), *nrd*J (vng1644) (ribonucleotide reductase α subunit), vng1642 (a conserved hypothetical halophile ORF adjacent to *nrd*J), *arc*A (vng6317), *arc*B (vng6315) and *arc*C (vng6316) (all of which are required for fermentation of arginine), *dbp *(vng2167) (coding for a eukaryote-like DNA binding protein of the superfamily I DNA and RNA helicases) and vng17 and vng261, small ORFs unique to *Halobacterium *sp. NRC-1 and with unknown functions.

**Figure 1:**
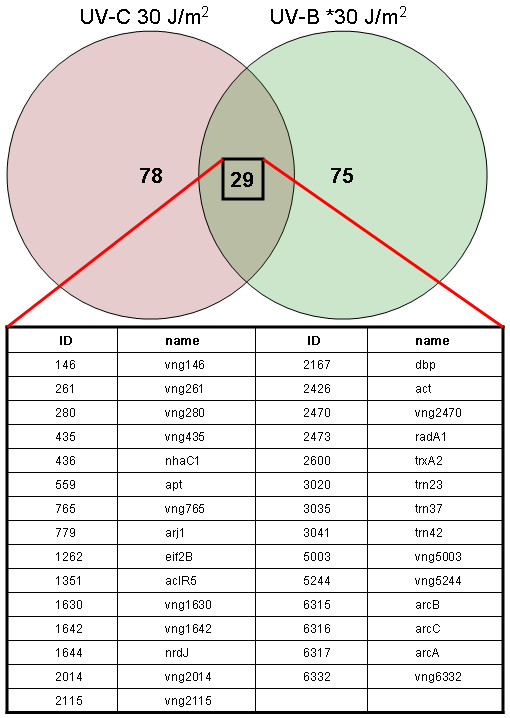
Diagram showing overlap between genes up-regulated 1.5-fold or more after irradiation with 30 J/m^2 ^UV-C and *30 J/m^2 ^UV-B (a dose of UV-B inducing an equivalent number of CPDs in DNA to 30 J/m^2 ^UV-C).

We compared the results of this experiment to our previously published 30 J/m^2 ^UV-C data and found that, of the 103 genes identified as up-regulated, 29 were also up-regulated in the 30 J/m^2 ^UV-C arrays (Figure 1 and Table 2).

**Table 2:** Genes up-regulated 1.5-fold or more by both UV-C (30 J/m^2^) and a damage-equivalent dose of UV-B

Gene ID	Gene name	Functional group	Predicted gene product	Fold increase **UV-C 30 J/m**^**2**^		Fold increase UV-B *30 J/m^**2**^	
				1 h	3 h	1 h	3 h
**146**	**vng146**	Unknown	NA	-1.02	**1.49**	-1.85	**2.01**
**261**	**vng261**	Unknown	NA	**2.20**	**2.09**	**1.85**	1.36
**280**	**vng280**	Unknown	NA	1.33	**1.47**	**1.66**	**1.54**
**435**	**vng435**	Unknown	NA	-1.11	**1.71**	1.44	**2.11**
**436**	***nha*C1**	Transport	Na+/H+ antiporter	1.19	**1.79**	1.23	**1.99**
**559**	***apt***	Nucleotide metabolism	Adenine phosphoribosyltransferase	**1.69**	**1.54**	**1.52**	**1.47**
**765**	**vng765**	Unknown	NA	**1.69**	**1.54**	**1.53**	**1.75**
**779**	***arj*1**	DNA metabolism	Archaeal RecJ-like exonuclease	1.35	**1.73**	**1.62**	**1.73**
**1262**	***eif*2B**	Translation	translation initiation factor eIF-2 subunit beta	**1.66**	**1.61**	1.25	**1.66**
**1351**	***acl*R5**	Transcription and regulation	Transcription regulator	**1.53**	**1.42**	**1.51**	**1.66**
**1630**	**vng1630**	Unknown	NA	**1.55**	**1.45**	**1.58**	1.40
**1642**	**vng1642**	Unknown	NA	**3.49**	**6.31**	**4.44**	**3.82**
**1644**	***nrd*J**	Nucleotide metabolism	Class II ribonucleotide reductase alpha subunit	**2.24**	**3.87**	**3.65**	**3.59**
**2014**	**vng2014**	Unknown	NA	**1.55**	1.19	**1.68**	**2.26**
**2115**	**vng2115**	Unknown	NA	**1.62**	**1.48**	**1.64**	**1.78**
**2167**	***dbp***	DNA metabolism	DNA binding protein eukaryotic-like	1.34	**1.86**	**2.17**	**1.57**
**2426**	***act***	Energy metabolism	Acyl-CoA thioester hydrolase	1.10	**2.46**	1.21	**1.66**
**2470**	**vng2470**	Unknown	NA	**1.46**	**1.56**	1.37	**1.60**
**2473**	***rad*A1**	DNA	Rad51/RecA recombinase	**8.80**	**8.14**	**9.32**	**6.74**
**2600**	***trx*A2**	Nucleic acid Metabolism	Thioredoxin	1.26	**1.56**	**1.55**	1.36
**3020**	***trn*23**	Translation	Leu-tRNA-CAA	**1.55**	1.43	1.14	**1.66**
**3035**	***trn*37**	Translation	His-tRNA-GTG	-1.38	**1.47**	**1.74**	**1.50**
**3041**	***trn*42**	Translation	Cys-tRNA-GCA	1.08	**1.80**	**1.55**	**1.76**
**5003**	**vng5003**	Unknown	NA	**1.50**	**1.68**	1.07	**1.58**
**5244**	**vng5244**	Unknown	NA	1.34	**1.77**	**1.46**	**1.61**
**6315**	***arc*B**	Amino acid metabolism	Ornithine carbamoyltransferase	**3.64**	1.25	**2.05**	1.01
**6316**	***arc*C**	Amino acid metabolism	Carbamate kinase	**6.66**	1.34	**2.63**	**1.46**
**6317**	***arc*A**	Amino acid metabolism	Arginine deiminase	**2.40**	**1.67**	**2.70**	1.01
**6332**	**vng6332**	Unknown	NA	**1.58**	**1.53**	1.34	**1.60**

Note: NA = not annotated

### Genes up-regulated in wild-type cells after *5 J/m^2 ^UV-B

**Figure 2:**
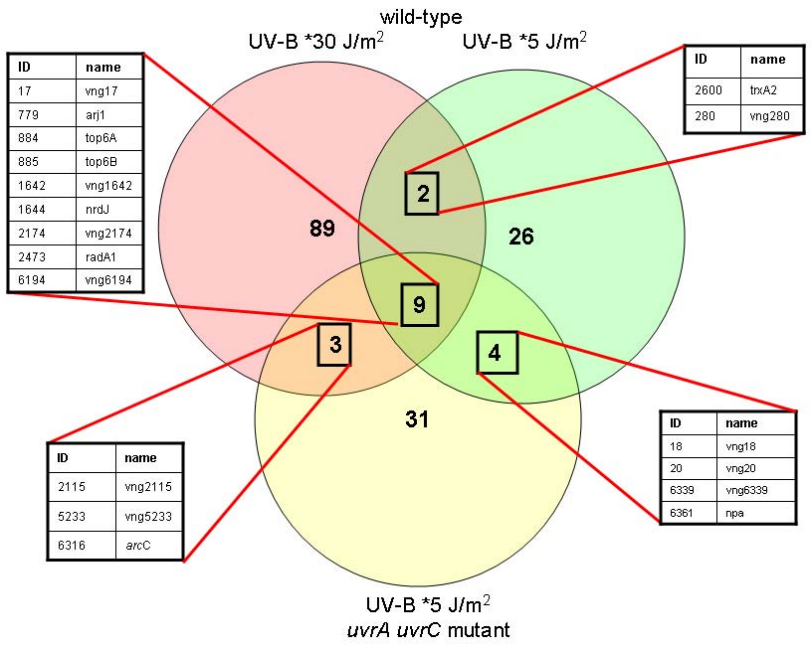
**Diagram showing the overlap between genes up-regulated 1.5-fold or more in the three UV-B experiments described in this work.** Asterisks indicate that irradiation of wild-type and a Δ*uvr*A Δ*uvr*C repair-deficient mutant were performed at a dose equivalent to a UV-C dose of 5 J/m^2 ^and irradiation of wild-type cells were performed at a dose equivalent to a UV-C dose of 30 J/m^2^.

At the lower UV-B dose, only 41 genes were significantly up-regulated in the wild-type strain. Of these, 11 were also up-regulated at *30 J/m^2 ^UV-B (Figure 2 and Table 3). These are the genes whose transcriptional control is most likely to be significant for the response to biologically relevant UV doses.

**Table 3:** Genes up-regulated 1.5-fold in UV-B experiment

			Fold increase		Fold increase		Fold increase	
			UV-B *30 J.m-2		UV-B *5 J.m-2		UV-B *5 J.m-2	
Gene			wild type		wild type		Δ*uvr*A Δ*uvr*C	
ID	name		1 h	3 h	1 h	3 h	1 h	3 h
**genes up-regulated in all three UV-B experiments**								
						
**17**	**vng17**	NA	**1.88**	**1.55**	**1.59**	**1.48**	**1.46**	**2.45**
**779**	***arj*1**	Archaeal RecJ-like exonuclease	**1.62**	**1.73**	1.34	**1.57**	1.23	**1.58**
**884**	***top*6A**	DNA topoisomerase VI subunit A	**1.48**	**1.58**	**1.57**	**1.82**	1.35	**1.68**
**885**	***top*6B**	DNA topoisomerase VI subunit B	**1.54**	**1.54**	1.31	**1.74**	1.23	**1.68**
**1642**	**vng1642**	Hypothetical protein VNG1642	**4.44**	**3.82**	**2.06**	**2.75**	**2.10**	1.25
**1644**	***nrd*J**	Class II ribonucleotide reductase alpha subunit	**3.65**	**3.59**	**1.87**	**2.53**	**1.67**	**2.53**
**2174**	**vng2174**	NA	**1.47**	**1.80**	1.40	**1.69**	-1.65	**1.92**
**2473**	***rad*A1**	RadA/RecA recombinase	**9.32**	**6.74**	**2.42**	**4.66**	**2.21**	**3.26**
**6194**	**vng6194**	NA	**1.80**	**2.20**	**1.88**	**2.44**	1.13	**4.04**

genes up-regulated only in wild type UV-B *30 J and UV-B *5 J experiments								
						
**280**	**vng280**	hypothetical protein VNG0280	**1.66**	**1.54**	**1.48**	**1.49**	1.14	1.40
**2600**	***trx*A2**	thioredoxin	**1.55**	1.36	**1.51**	1.38	1.14	-1.37

genes up-regulated only in UV-B *5 J experiments, wild-type and mutant								
						
**18**	**vng18**	NA	1.20	1.26	1.27	**1.50**	**1.57**	**3.03**
**20**	**vng20**	NA	1.10	1.04	1.10	**1.84**	-1.10	**1.70**
**6339**	**vng6339**	NA	1.10	-1.05	**1.99**	1.02	1.44	**2.30**
**6361**	***npa***	Predicted transposase	1.16	1.03	**2.10**	**2.73**	**1.66**	**3.01**

genes up-regulated only in wild type UV-B *30 J and UV-B *5 J mutant experiments								
						
**2115**	**vng2115**	NA	**1.64**	**1.78**	1.43	1.19	1.03	**1.60**
**5233**	**vng5233**	NA	-1.05	**1.57**	-1.08	1.08	1.11	**1.46**
**6316**	***arc*C**	Carbamate kinase	**2.63**	**1.46**	1.35	1.01	**1.79**	1.00

Note: NA = not annotated

### Genes up-regulated after *5 J/m^2 ^UV-B in a repair-deficient mutant

In addition to analysing the transcriptional response to UV in wild-type *Halobacterium *sp. NRC-1 cells, we measured the response to *5 J/m^2 ^UV-B in a Δ*uvr*A Δ*uvr*C knockout strain which lacks the capacity for nucleotide excision repair [10] so that we could examine responses in the absence of repair (and, presumably, the persistence of DNA damage). NRC-1 cells are able to remove UV damage by excision repair relatively rapidly [17] and most photoproducts are repaired within 3 hours after irradiation. So we anticipated that, if the response was related to amount of damage in DNA, the transcriptional response to UV in a repair-deficient mutant might resemble the response to a higher dose in wild-type cells. However, we found that the response to a dose of *5 J/m^2 ^UV-B was very similar in both the wild-type and repair-deficient mutant. The total number of genes up-regulated was very similar, 41 and 47 respectively, and there was considerable overlap, with 13 genes up-regulated in common (Figure 2). The fold changes were also similar to the wild-type at the same dose and lower than the fold changes seen after the higher dose, with the possible exception of *arcC *(see Table 3). This suggests that the nature of the transcriptional response does not simply depend on the number of DNA photoproducts present in the DNA.

### Comparison of all UV-B and UV-C arrays

Table 4 shows the fold-changes for selected transcripts in the five experiments we have carried out, irradiating wild-type and mutant cells with various doses of UV-C and UV-B [31]. The table highlights the fact that some genes, including *rad*A1 (vng2473), *nrd*J (vng1644), vng1642, *arj*1 (vng779) and *trx*A2 (vng2600) were up-regulated by all or most UV-irradiation regimes. Other genes, notably *hjr *(vng2252), vng261, vng1800, *rfa*3 (vng2160), and the *arc*ABC genes, were up-regulated only by higher doses or only by short-wave UV. Most interestingly several genes – *npa *(vng6361), vng17, vng6359 (which is similar to vng17 and is located directly upstream of *npa*), *top*6A (vng884) and *top*6B (vng885) were significantly up-regulated only by lower doses or by UV-B.

**Table 4:** Transcriptional response of selected genes in UV-C [26] and UV-B microarray experiments

Gene ID & Name		UV-C NRC1		UV-C NRC1		UV-B NRC1		UV-B NRC1		UV-B uvrA uvrC	
		**70 J/m**^**2**^		**30 J/m**^**2**^		*30 J/m^**2**^		*5 J/m^**2**^		*5 J/m^**2**^	
		1 h	3 h	1 h	3 h	1 h	3 h	1 h	3 h	1 h	3 h
2473	***rad*A1**	**9.35**	**7.35**	**8.80**	**8.14**	**9.32**	**6.74**	**2.42**	**4.66**	**2.21**	**3.27**
1642	**vng1642**	**5.32**	**6.55**	**3.49**	**6.31**	**4.44**	**3.82**	**2.06**	**2.75**	**2.09**	**1.25**
1644	***nrd*J**	**3.20**	**4.06**	**2.24**	**3.87**	**3.65**	**3.59**	**1.87**	**2.53**	**1.67**	**2.53**
											
2383	***nrd*A**	**-1.46**	**2.16**	**1.01**	**2.01**	**1.03**	**1.07**	**1.17**	**1.19**	**1.36**	**1.13**
6317	***arc*A**	**1.25**	**1.05**	**2.40**	**1.17**	**2.70**	**1.01**	**-1.03**	**1.01**	**1.22**	**-1.05**
6315	***arc*B**	**1.43**	**-1.00**	**3.64**	**1.25**	**2.05**	**1.01**	**-1.13**	**1.08**	**1.03**	**-1.17**
6316	***arc*C**	**2.55**	**1.17**	**6.66**	**1.34**	**2.63**	**1.46**	**1.35**	**1.01**	**1.79**	**1.00**
											
2167	***dbp***	**2.04**	**1.82**	**1.34**	**1.86**	**2.17**	**1.57**	**1.18**	**-1.12**	**1.01**	**-1.01**
261	**vng261**	**1.66**	**2.21**	**2.19**	**2.09**	**1.85**	**1.36**	**1.21**	**1.08**	**1.26**	**-1.00**
1800	**vng1800**	**1.55**	**2.72**	**2.23**	**2.58**	**1.27**	**1.34**	**1.27**	**1.15**	**1.14**	**1.15**
2080	***blo*B**	**2.00**	**2.10**	**2.10**	**1.83**	**1.24**	**1.43**	**1.22**	**1.22**	**1.10**	**1.34**
2160	***rfa*3**	**1.51**	**1.52**	**1.56**	**1.34**	**1.02**	**1.19**	**-1.23**	**1.19**	**-1.08**	**1.18**
2252	***hjr***	**1.31**	**1.68**	**1.28**	**1.48**	**1.20**		**-1.02**	**1.16**	**-1.10**	**-1.49**
											
779	***arj*1**	**1.49**	**1.31**	**1.35**	**1.73**	**1.62**	**1.73**	**1.34**	**1.57**	**1.23**	**1.58**
2600	***trx*A2**	**1.75**	**1.83**	**1.26**	**1.56**	**1.55**	**1.36**	**1.52**	**1.38**	**1.12**	**-1.37**
2115	**vng2115**	**2.32**	**1.99**	**1.62**	**1.48**	**1.64**	**1.78**	**1.43**	**1.19**	**1.03**	**1.60**
280	**vng280**	**1.62**	**1.64**	**1.33**	**1.47**	**1.66**	**1.54**	**1.48**	**1.49**	**1.14**	**1.40**
											
17	**vng17**	**1.46**	**1.15**	**1.18**	**1.16**	**1.88**	**1.55**	**1.59**	**1.48**	**1.46**	**2.45**
884	***top*6A**	**1.15**	**1.37**	**1.17**	**1.35**	**1.48**	**1.58**	**1.57**	**1.82**	**1.35**	**1.68**
885	***top*6B**	**1.50**	**1.32**	**1.12**	**1.21**	**1.54**	**1.54**	**1.31**	**1.74**	**1.23**	**1.68**
6361	***npa***	**1.04**	**-1.02**	**1.07**	**-1.09**	**1.17**	**1.03**	**2.10**	**2.73**	**1.66**	**3.01**

Only four genes were up-regulated 1.5-fold or more in response to all of the doses of UV-C and UV-B we have used, at at least one time point. These are *rad*A1 (vng2473), *arj*1 (vng779), *nrd*J (vng1644) and vng1642.

### Confirmation of up-regulation with quantitative real time PCR

**Figure 3:**
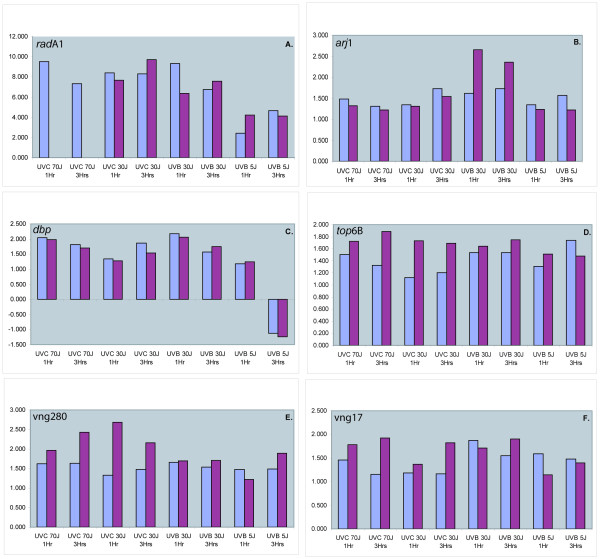
Histograms showing the fold changes in transcripts from microarray data (blue) and confirmation by qRT-PCR (maroon) of six selected genes: A. *rad*A1 (vng2473), B. *arj*1 (vng779), C. *dbp *(vng217), D. *top*6B (vng885), E. vng280, F. vng17.

Six genes, including *rad*A1 (vng2473), were selected for confirmation of the up-regulation noted from microarray data using qRT-PCR (Figure 3). The results agree well with the microarray data, for all doses and all wavelengths, and confirm that these genes are indeed up-regulated by UV in most cases. In a few cases the RT-PCR results do not agree quantitatively with the microarray data; in these instances, qRT-PCR showed somewhat greater up-regulation than was evident from the microarray data. The most dramatically up-regulated gene, *rad*A1, is up-regulated 9.7-fold, three hours after 30 J/m^2 ^UV-C, 7.6-fold after an equivalent dose of UV-B and over 4-fold after the much lower UV-B dose (*5 J/m^2^).

### A motif common to the promoter regions of five UV-B up-regulated genes

**Figure 4:**
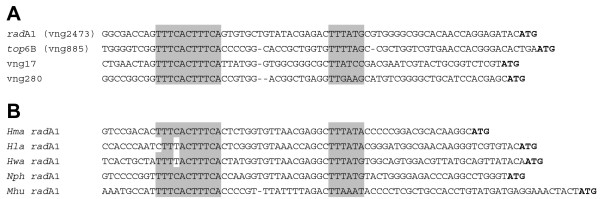
**(A) Sequence alignments of promoter regions of four genes up-regulated by UV-B in *Halobacterium *sp. NRC-1, showing that they share an 11-base pair sequence motif upstream of the promoter.** (B) Sequence alignments of promoter regions of *rad*A genes of other archaea containing an identical or similar 11-base-pair motif. The 11-base pair motif and putative TATA-boxes are highlighted by shading. *Hma*, *Haloarcula marismortui*; *Hla*, *Halorubrum lacusprofundi*; *Hwa*, *Haloquadratum walsbyi*; *Nph*, *Natronobacterium pharaonis*; *Mhu*, *Methanospirillum hungatei*.

Since *rad*A1 (vng2473) was consistently the most highly up-regulated gene in all our experiments, we examined its promoter region and noticed a striking sequence motif, TTTCACTTTCA, with an internal 5 bp repeat (TTTCA), located about 50 bases upstream of the start codon. A findpatterns search of the *Halobacterium *sp. NRC-1 genome revealed seven matches of this 11-base sequence. Four were in UV-B up-regulated genes (*rad*A1, vng280, *top*6B, and vng17) and one was on a non-coding strand. Alignments of the promoters of these genes are shown in Figure 4A. A proviso is that the alignment in the figure uses the second ATG in the vng280 ORF as the translational start codon rather than the first predicted using Glimmer in the genome sequence [12]. Interestingly, a near-match (TTTTACTTTCA) to the 11-base pair motif is found 52–62 bases upstream of the start codon of *npa*, a putative transposase gene, which is also up-regulated after UV-B irradiation. A similar motif is found located in the upstream regions of *rad*A genes in other halophilic archaea and, interestingly, *Methanospirillum *(Figure 4B). It is not found in any of the *rad*A2 (vng1665) promoter regions examined (not shown).

## Discussion

Previous genomic transcriptional analyses in the archaea have shown large numbers of genes to be up-regulated after irradiation with high doses of UV-C and experiments by different groups have shown considerable differences in the genes identified [11,25,26]. The use of low doses of UV-B has enabled us to focus on a smaller set of genes, whose transcriptional response is more likely to be biologically and environmentally significant than the genes identified previously. These low-dose experiments have confirmed the upregulation of *rad*A1 (vng2473) previously identified in high dose UV-C experiments and revealed the up-regulation of several genes that were not, including *top*6B (vng885), vng17 and *npa *(vng6361). We have shown that *top*6B and vng17, as well as vng280, all share a common motif with *rad*A1 (vng2473) in the promoter region which seems very likely to be involved in transcriptional regulation in response to DNA damage. A nearly identical motif is also present upstream of *npa*.

The proteins encoded by these genes may have related functions in the cell's response to UV radiation. RadA1 is likely to play a major role in resolving stalled replication forks and/or promoting repair [32-34] and it is likely to be required in large amounts because it coats single-stranded DNA to form nucleoprotein filaments [35], hence the greatest fold-induction observed after UV radiation. *top*6A (vng884) and *top*6B (vng885) code for DNA topoisomerase VI subunits A and B. The little-studied archaeal topoisomerase VI enzymes are members of the topoisomerase IIB family and have been shown to be important in both *Sulfolobus *and halophilic archaea [36]. They have ATP-dependent nicking-closing activity as well as ability to generate double-strand breaks and they are able to release positive supercoils that are formed ahead of replication forks and during transcription [37,38]. It is likely that they are involved in processing stalled forks in UV-damaged DNA in *Halobacterium*. *arj*1 (vng779), which is up-regulated by all UV-B doses examined, encodes a RecJR-like protein, so, by analogy to *E. coli *RecJR it, too, is likely to be involved in recovery of DNA replication at stalled forks, possibly by making DNA lesions at stalled forks accessible for repair [39]. We do not know the functions of the vng17 and vng280 gene products. If these genes are indeed up-regulated because of their role in recovery of DNA replication, we speculate that the reason why they are not significantly up-regulated after high UV doses is that high doses may largely halt initiation and/or elongation of DNA replication [40,41]. Therefore, after high doses of UV irradiation, there are fewer replication forks that become blocked. However, the precise roles for these genes must await further experimentation, including genetic knockouts and perturbations.

After the higher dose of UV-B, we observed up-regulation of *arc*A, *arc*B and *arc*C, though only at the earlier time after irradiation (Table 5); this is similar to the response we saw after UV-C irradiation at 30 J/m^2 ^and 70 J/m^2 ^[26]. We do not see these genes up-regulated after low UV-B doses, except for slight up-regulation of *arc*C in the repair-deficient mutant and we do not know the significance of this response. We suggested in an earlier report that up-regulation of these genes may reflect a demand for rapid supply of ATP during periods of DNA-damage repair [26] or it may be a more general stress response.

**Table 5:** Primers and Taqman probes used for q-PCR

Primer name	Sequence 5'-3'	Size (bp)	Accession numbers
RadA1-rtF	ACACCCTCACGGAGCTCGT	77	GI:10581871
RadA1-rtR	CATCTGGTGGTTGGAGTTGAAG		
RadA1-probe	6-FAM-TCCTGGACAAGATCCACGTCGCG-BHQ1		
Vng17-rtF	TGTCACGGTGATTGGTTTCG	92	GI:10579665
Vng17-rtR	AAGTCTGCAGAGTTTCTGCATCG		
Vng17-probe	6-FAM-CACGACCTCGGCACGTGGCTAGT-BHQ1		
Vng280-rtF	CAGAATGGCGTCCTCGTCGT	128	GI:10579913
Vng280-rtR	GGACGCAGTTCGAACTCCTCTC		
Vng280-probe	6-FAM-TACGCGCCCACCGTGCTGACCG-BHQ1		
Top6B-rtF	TCCACGACTACATCAAACACACG	89	GI:10580449
Top6B-rtR	GCGCTCTGATTTGAGCTCG		
Top6B-probe	6-FAM-TCGTGAACCCACACGCCCGCAT-BHQ1		
Vng0779-rtF	ATGAGCGAGGCCCTCGATTAC	80	GI:10580354
Vng0779-rtR	ACGTTCAGGATGTCCGCGAT		
Vng0779-probe	6-FAM-TACATGCTCCGGTACGACCACGGCA-BHQ1		
Dbp-rtF	GCCACCTCTCGCTGGTCG	108	GI:10581584
Dbp-rtR	CGAGCGTGTCGTAGAGGTCG		
Dbp-probe	6-FAM-TACACGTCTGCGCAGCTCGCTGC-BHQ1		
Eef2-rtF	ACGAAAGAAGATTGTCGAACAGTG	110	GI:10582035
Eef2-rtR	TGTCAGTGAGGGTGGTTTTTCC		
Eef2-probe	JOE-AACGGCTGATGGACAACCCGGAGC-BHQ1		

The level of up-regulation of *rad*A1 that we see in *Halobacterium *sp. NRC1 is similar to that reported for the archaeal mesophiles, *Methanococcus maripaludis *and *Methanococcus voltae*. Reich *et al*. [27], using Northern blot analysis of transcripts and Western blots to study RadA protein levels, found that *rad*A transcription was up-regulated, and RadA protein levels increased, in the four archaea studied. The up-regulation was greater (about 6-fold after a UV dose of 50 J/m^2^) in the mesophiles, *Methanococcus maripaludis *and *Methanococcus voltae*, than in the thermophiles (about 2-fold), *Sulfolobus solfataricus *and *Methanococcus jannaschii*. A recent transcriptomic study using microarrays after a range of UV doses did not show significant up-regulation of *rad*A in *Sulfolobus solfataricus *[28], possibly reflecting the low level of the response or, perhaps, the use of different growth conditions.

It is seems likely that the 11-bp motif, TTTCACTTTCA, that we have identified upstream of the start codon is involved in regulation of the genes that share it – *rad*A1 (vng2473), vng17, vng 280, and *top*6B (vng885) – and it may be the binding site for a transcriptional regulator. It is interesting that three of the genes that have this motif were not originally identified in our high-dose UV-C experiments but that they were all up-regulated after UV-B exposure. Neither *top*6B, vng280, nor vng17 is up-regulated to as high a level as *rad*A1 (Figure 3 and Table 4). However we are currently carrying out a detailed study of the *rad*A1 promoter region and have found that the *rad*A1 upstream region contains an additional putative regulatory sequence that is not present in the other three genes (unpublished).

Interestingly, in *Sulfolobus solfataricus*, a crenarchaeon, SSO0777, which is a paralogue of the *rad*A gene, is regulated in response to DNA damage, by the activator Sta1, which binds within the sequence ATTTTTTATTTTCACATGTAAGATGTTTATT [42]. There is no obvious homologue of Sta1 in *Halobacterium*, however, and it is not clear whether the two systems have common evolutionary origins. The *Halobacterium *11-bp motif, TTTCACTTTCA, is similar to the 5' half of this repeat, with one copy of the 5-bp internal duplication present.

Our findings suggest that experiments employing high UV-C doses are not a good model for the response to environmentally relevant UV radiation. Strikingly, none of the four genes that were up-regulated in response to all of the doses of UV-C and UV-B we have used [*rad*A1 (vng2473), *arj*1 (vng779), *nrd*J (vng1644) and vng1642] was found to be significantly up-regulated in a previous study by Baliga *et al*. in which a very high dose of UV was used (see Table 1) [11]. A similar observation has been made in *Schizosaccharomyces pombe*, where transcription of *rhp*51, the *rad*A homologue, was up-regulated after low doses of UV-C but not after high doses (200 J/m^2 ^and above) [43] and it was suggested that extensive DNA damage and blocking of DNA replication prevented up-regulation. In *Saccharomyces cerevisiae*, too, high doses of UV have not been informative. Genes shown to play a role in survival of UV irradiation (with deletion mutants that were sensitive to UV) failed to correlate with genes that were transcriptionally up-regulated by a high dose of UV-C (200 J/m^2^) [44], so studies of transcriptional response to high doses of UV-C could not identify genes involved in surviving UV irradiation.

One of the distinguishing features of the current study is that we used UV-B light, in contrast to short-wave UV-C commonly used in laboratory studies of UV damage. Whilst it is true that the photoproducts induced by UV-C, UV-B and sunlight are broadly similar and that they are all repaired by nucleotide excision repair, there are significant differences in the damage induced by different UV light sources. Perdiz et al. [1] measured the proportions of the three major types of photoproduct formed in DNA on exposure to different sources of UV light – a UV-C lamp emitting at 254 nm, a broad-band UV-B lamp and a solar simulator. They found that the proportions of cyclobutane pyrimidine dimers (CPDs) to 6-4 photoproducts (6-4 pps) to Dewars induced in DNA were 1.0:0.25:0 for the UV-C lamp, 1.0:0.12:0.014 for the broad-band UV-B lamp and 1.0:0.18:0.06 for the solar simulator [1]. These results showed that UV-B, though not identical in its effects to sunlight, is a closer model than UV-C because both sunlight and UV-B induce a significant number of Dewars as well as inducing relatively fewer 6-4pps. They also measured repair of the three types of photolesion and found that both CPDs and Dewars are repaired much more slowly than 6-4 pps [1].

We have compared the doses used in published microarray studies to the UV doses found in sunlight (Table 1). These are, inevitably, approximations since the UV doses and wavelengths in sunlight vary with latitude, altitude, time of day and local conditions. The figures we have used are based on the maximum number of CPDs induced by sunlight during a whole day's exposure, measured by Wilhelm et al. using a DNA dosimeter, at equatorial latitudes off the coast of South America [29] and Visser et al., also using a DNA dosimeter, off the south coast of Curacao (12 ° 07' N) [30].

Finally, *Halobacterium *sp. NRC-1 has also been the subject of studies with ionizing radiation from both gamma and electron beam sources. In one study conducted by DeVeaux et al [45], two highly radiation resistant *Halobacterium *mutants were reported which, with a LD_50 _of nearly 12 KGy, are even more resistant than *Deinococcus radiodurans*, previously the most radiation resistant organism known. The mutants upregulated the expression of *rfa3 *and two transcriptionally-linked downstream genes, which are also inducible after high UV-C exposure. The ability of *Halobacterium *to survive both ionising and non-ionising radiation is a remarkable property of these species and suggests that more detailed investigations will provide a much better understanding of the DNA repair and replication systems operating in these model Archaea.

## Methods

### Culture conditions and UV-irradiation

*Halobacterium *sp. strain NRC- 1 and the Δ*uvr*A Δ*uvr*C mutant, were grown in the dark, at 37°C, in an orbital shaker-incubator at 225 rpm, under aerobic conditions to early exponential growth phase (OD_600 _0.19–0.23) in complete medium, CM [46]. 50-ml cultures were grown up in triplicate for each time point. For irradiation, cultures were transferred individually into pre-warmed plastic boxes and irradiated in the dark, in CM^+ ^medium with gentle agitation, using two unfiltered FS20 fluorescent tubes as the UV-B source. In order to compare the transcriptional profiles after UV-B irradiation with our previous studies, in which we irradiated with 30 and 70 J/m^2 ^UV-C, from a mercury vapour lamp emitting at 254 nm, we irradiated plasmid DNA and measured cyclobutane dimers (i.e. sites sensitive to nicking by micrococcal UV-endonuclease [47]). The number of cyclobutane pyrimidine dimers induced in plasmid DNA by the UV-B lamp in 30 sec was shown to be equal to the number induced by 5 J/m^2 ^UV-C. An equivalent UV-B dose to 30 J/m^2 ^UV-C was administered by irradiating for 3 minutes. UV-B doses are referred to as 'damage-equivalent' doses. For post-UV incubation, cultures were returned to the original warmed flasks and incubation was continued at 37°C in the dark. We avoided changing the medium, so as to avoid any additional stress caused to the cells by harvesting and changing media.

### Primer and fluorescence probe design

Six genes were selected for qRT-PCR fold change validation. These were *rad*A1 (vng2473) (DNA repair and recombination protein RadA1, RAD51/RecA homologue), vng17 (hypothetical protein), vng280 (hypothetical protein), *top*6B (vng885) (DNA topoisomerase VI subunit B), *arj*1 (vng779) (*rec*J-like exonuclease), and *dbp *(eukaryote-like DNA binding protein). The housekeeping gene *eef2 *(vng2654) (translation elongation factor eEF-2) was used as an internal control. Sequences were retrieved from the NCBI GenBank database with the accession numbers shown in Table 5. Primers and probes were designed using Primer Expression™ version 2.0 software (PE Applied Biosystems, CA). Taqman probes were labelled with either 6-FAM or JOE and paired with Black Hole Quenchers^® ^(BHQ1). All primers and Taqman^® ^probes were synthesised by Biomers.net (Germany). Primers and PCR product sizes in this study are shown in Table 5.

### cDNA synthesis for RT-PCR

cDNAs were reverse transcribed with M-MLV Reverse Transcriptase, RNase H Minus, Point Mutant (Promega, USA) as described in the manufacturer's instructions. Briefly, 2 μg of DNase-treated total RNA was mixed with 7.5 μM specific reverse primers (both query gene and *eef*2) and incubated for 5 min at 70°C, following by fast cooling on ice for another 5 min. The mixture was added to a final concentration of 1× M-MLV RT reaction buffer, 0.5 mM dNTPs, 6.0 U M-MLV RT (H-) enzyme, 0.32 U RNaseOUT™ (Invitrogen, USA) and finally made up to 25 μL total volume with RNase-free water and the mixture was incubated for 1 hour at 55°C. The enzyme was inactivated by heating for 15 min at 70°C.

### Quantitative real time PCR (qRT-PCR)

qRT-PCR was performed on an ABI Prism 7500 sequence detector (PE Applied Biosystems, CA). Each UV dose or time point sample was prepared in three biological replicates, each with triplicate qPCR reactions. The PCR reaction mixture contained a final concentration of 1× FastStart Taqman^® ^Probe Master (Rox) (Roche, Germany), 280 mM Taqman^® ^probe, 300 mM forward and reverse primers, 5 μL of 100× diluted cDNA, made up to 25 μL total volume with RNase-free water. Two different reactions were prepared for *eef*2 and the query gene and both were quantified in the real time PCR machine within the same run. The PCR amplification programme was: enzyme activation at 95°C for 10 min following by 35 cycles of denaturation at 95°C 1 min, and annealing at 60°C for 30 sec. The results were analysed using 7500 SDS version 1.3 (PE Applied Biosystems, CA). All the calculations of relative fold change were done against individual external standard curves.

### Microarray procedures

Relative mRNA levels were determined by parallel two-colour hybridization to oligonucleotide (60-mer) microarrays representing 2,677 open reading frames (ORFs) representing 99.9 % of *Halobacterium *sp. NRC-1 ORFs [48]. Total RNA was isolated from 50-ml cultures immediately after harvesting using Agilent Total RNA isolation kit (Agilent, USA) and DNA was hydrolysed using amplification grade DNase (Sigma, UK). In order to minimize biological noise, RNA preparations from three cultures grown and irradiated under identical conditions were pooled to equal parts for cDNA synthesis. cDNA was prepared from 7 μg total RNA with Super Script III reverse transcriptase (Invitrogen, UK) and Cy3- or Cy5-dCTP (Amersham Biosciences, UK). Performance of duplicate experiments in which dyes were swapped during synthesis to account for labeling differences was not required. Previous results showed that differences in the relative intensity of the channels could be adjusted for by intensity-dependent LOWESS [31]. cDNA preparations were purified after alkaline hydrolysis of RNA on Qiagen mini-elute columns (Qiagen, UK). The labeled cDNA targets were mixed with hybridization buffer and control targets (Agilent, USA), and hybridized to microarray slides, assembled into a hybridization chamber (Agilent, USA), for 17 h at 60°C in the dark. Post hybridization, the slides were washed as described and scanned for the Cy3 and Cy5 fluorescent signals with an Agilent DNA-microarray scanner (Model no. G2565BA). Image processing and statistical analysis were carried out using Agilent Feature Extraction Software Version 7.1 as described previously [31]. Log ratios for each feature were calculated and the significance of the log ratio was assessed by calculating the most conservative log ratio error and significance value (p-value) using a standard error propagation algorithm (Agilent) and a universal error model (Rosetta Biosoftware). The illuminant intensity, log_2_(x) value, and standard deviation of the log_2_(x) value were calculated for the normalized red and green probe values for each gene in each microarray. The illuminant intensity was calculated through the logarithm of the geometric mean of *Cy*5 and *Cy*3 processed signal intensities as previously described [48]. Standard deviations for sample means of log_2_(x) ratios were calculated and changes in transcript levels were considered significant if they were changed about 1.5-fold or more using a linear transform function.

## Competing interests

The authors declare that they have no competing interests.

## Authors' contributions

SJM designed the UV irradiation experiments in consultation with SD, analyzed the data, and drafted the manuscript. WLN designed and carried out the real time PCR experiments, assisted with UV irradiation, RNA preparation, cDNA labelling and analysis of the data. IB assisted with experimental design and conducted the UV irradiation, RNA preparation, and cDNA labelling. DJC constructed and characterised the Δ*uvr*A Δ*uvr*C double deletion mutant. PD conducted the DNA microarray hybridization, data processing and analysis and assisted extensively with preparation of the figures, tables, and manuscript text. SD assisted with experimental design, data interpretation and finalising the manuscript. All authors read and approved the final manuscript.
